# When are people more open to cheating? Economic inequality makes people expect more everyday unethical behavior

**DOI:** 10.1371/journal.pone.0294124

**Published:** 2024-02-21

**Authors:** Anita Schmalor, Adrian K. Schroeder, Steven J. Heine

**Affiliations:** 1 University of British Columbia, Vancouver, British Columbia, Canada; 2 University of Toronto, Toronto, Canada; Private University Schloss Seeburg: Privatuniversitat Schloss Seeburg, AUSTRIA

## Abstract

Economic inequality has been found to be associated with increased unethical behavior and an increased acceptance of unethical behavior. In this paper we explored whether higher amounts of perceived inequality lead to an increase in the expectation of unethical behavior. We tested whether people would say that they themselves would engage in more unethical behavior in a context of high compared to low inequality. We find evidence for this hypothesis in 3 of 4 studies (n = 3,038). An internal meta-analysis shows a small but significant effect. Such increased expectations that oneself will behave unethically likely has consequences for societal trust and functioning.

## Introduction

Societies vary considerably in their extent of economic inequality, and, in recent years, economic inequality has increased across much of the globe [e.g., 1, 2] and is expected to continue to increase [3]. There has been growing interest in how inequality shapes people’s psychology. One question of interest is how inequality affects ethical behaviors [e.g., 4–6]. Previous research suggests that higher inequality is associated with an increase in people’s own unethical behaviors [e.g., 4, 6–9; see 10 for a review]. Furthermore, correlational research finds that objective indicators of economic inequality are associated with such unethical behaviors as increased corruption [11, 12], tax evasion [13], higher crime and homicides rates [14, 15]; and students from more unequal US states were found to conduct more web search queries with terms related to academic dishonesty [6].

While the relations between inequality and various forms of unethical behaviors appear to be fairly robust, it is unclear whether people are aware of this relation. In the present studies we tested whether people *expect* that they would behave more unethically (as past research suggests) in high inequality contexts or whether their expectations remain unchanged. We reasoned that if people are sensitive to changes in inequality, they may adjust their expectations around unethical behavior accordingly. Therefore, we both manipulated and assessed people’s perceptions of inequality and tested how it affects their expectations of everyday unethical behavior.

Economic inequality refers to the extent to which economic resources such as income or wealth are distributed unequally within a society. The greater the amount of inequality, the greater the disparity in income or wealth among people in that society [16].

Past research has posited a relation between economic inequality and heightened competition between people [17, 18]. These findings are typically seen to be the product of what occurs when resources (i.e., income or wealth) are concentrated among people at the top of the distribution [but see 19]. Under such circumstances, climbing or falling down the social ladder can make a marked difference in one’s economic standing. On the other hand, when inequality is low, rising or falling in the ranks makes relatively less difference in terms of one’s economic situation. As a consequence, when inequality is high, people are more competitive [16, 18], and see competitive and individualistic behavior as more normative and likely to occur [20–22]. Similarly, when inequality is higher people are more willing to take risks in an effort to get ahead [23]. Furthermore, research shows that when people perceive more inequality, they become more self-focused and attend less to the emotions of others [24]. Taken together, this past research leads us to predict that when people perceive more inequality, they will become more likely to expect others and themselves to act more unethically. In line with this, recent research showed that when perceived and actual levels of inequality were higher, people found unethical behavior of others to be more acceptable [25].

In the present research, we focus on people’s expectations of everyday unethical behaviors, such as keeping extra change instead of returning it to the clerk or keeping a pair of pants that was delivered to the wrong address. We define unethical behavior here as everyday transgressions that violate social norms, although they aren’t necessarily against the law [e.g., 25–27]. Much research has focused on the effects of inequality on more severe forms of unethical behaviors such as crime [e.g., 4] or corruption [11]. If people expect that benign everyday unethical behavior is more common in unequal situations, it may shape their view of people as more selfish more generally, deteriorate trust and undermine societal functioning.

We test whether people expect that they themselves will engage in more unethical behavior when they perceive inequality to be high as opposed to low. Past research shows that people find unethical behavior more acceptable when they perceive inequality to be high [25]. This shift in acceptability suggests that it is possible that people are more likely to say they would engage in unethical behaviors too.

In sum, we hypothesized that perceptions of higher economic inequality would lead people to say that they themselves would engage in more unethical behavior. We test this hypotheses across 4 studies (3 of which were pre-registered) utilizing 3 different means of manipulating/assessing perceptions. In contrast to some of the past literature, we assessed the impact of economic inequality by using both experimental manipulations and correlational data.

For all studies, we report how we determined our sample size, all data exclusions, and all measures in the study. We pre-registered the hypotheses, measures, sample size, analyses, and exclusion criteria for Studies 1, 2b, and 3. The procedures of all studies were approved by our university’s behavioral research ethics board. Participants provided informed consent.

## Study 1

In Study 1, we manipulate perceived inequality by asking participants to imagine living in one of two societies which varied in terms of their inequality. We then assessed their own predictions of whether they would engage in a variety of different unethical behaviors in a vignette task. We pre-registered the hypothesis, measures, exclusion criteria, and analyses on the OSF [https://doi.org/10.17605/OSF.IO/WJ276].

### Participants

We solicited a convenience sample of Americans on CloudResearch [28]. A sample of 800 participants allows us to detect an effect of *d* = .20 at an alpha of .05, with 80% power in this between-groups design. To be above this number we pre-registered to collect data from 1040 participants. After excluding participants who failed any of the pre-registered attention checks, our final sample consisted of 907 participants (*M* age = 38.86; 62% female; 75% Caucasian, 9% African American, 16% other). A sensitivity power analysis for a two-tailed independent samples t-test with an alpha set to 0.05, 80% power and a sample size of 451 in the low, and 455 in the high inequality conditions [calculated in G*Power; 29] shows that the minimum effect size for these parameters is *d* = 0.186.

### Measures

#### Economic inequality

Participants read that they were to imagine living in Bimboola, a hypothetical society [see 30]. They learned that Bimboola has three income tiers and that they would be randomly assigned to one of them [see 31]. In the low inequality condition (Bimboolean Dollars 30,000; 40,000; 50,000), the income tiers had lower variability than in the high inequality condition (Bimboolean Dollars 3,000; 40,000; 77,000). The mean income per condition was the same, and all participants were assigned to the second income tier. Thus, only the level of inequality varied between conditions. To strengthen the manipulation, participants also had to choose a house, car, and vacation spot for their new life. For each category they saw three different options that people with high, middle, and low incomes could choose from. While all participants had to choose from the same options for the second tier, they saw different options for people of low and high incomes in the two different conditions.

#### Unethical behavior

Participants read 10 scenarios in which they engaged in unethical behavior they benefitted from (e.g., “You work in a fast-food restaurant in downtown Bimboola. It’s against policy to eat food without paying for it. You came straight from a doctor’s appointment and are therefore hungry. Your supervisor isn’t around, so you make something for yourself and eat it without paying.”) and had to indicate how likely they would be to engage in the behavior on a 7-point Likert scale from “extremely unlikely” to “extremely likely” (*M* = 3.43, *SD* = 1.23, α = .85; see SOM for a list of all items). The 10 scenarios are an adaptation of 8 scenarios that were used in previous research to assess people’s propensity to behave in ways that benefit them in some way but violate moral principles [32, 33]. The scenarios have been validated in previous research in different ways: independent business experts rated the scenarios as violation of ethical standards. Moreover, agreement with engaging in the behaviors describes was correlated with different forms of unethical behavior [33]. We adapted the scenarios to fit within the context of the hypothetical society participants were asked to imagine living in.

### Results

To test whether the manipulation was successful, we created a mean score of two questions asking participants the extent that the fictitious society of “Bimboola” was unequal on a 9-point scale where higher values indicate more inequality. Participants in the high inequality condition (*M* = 7.05, *SD* = 1.37) perceived Bimboola to be significantly more unequal than participants in the low inequality condition (*M* = 4.06, *SD* = 1.56), *b* = 2.98, *p* < .001, 95%CI = [2.79, 3.17], *d* = 2.04. Next, we tested our pre-registered hypothesis. Participants in the high inequality condition (*M* = 3.51, *SD* = 1.27) were marginally more likely to say they would engage in a variety of everyday unethical behaviors than participants in the low inequality condition (*M* = 3.35, *SD* = 1.18), *b* = 0.16, *p* = .057, 95%CI = [-0.004, 0.32], *d* = 0.13. We also pre-registered to conduct these analyses with ethnicity, age, and gender as covariates. The results are statistically significant (*p* = .023) with these covariates and are reported in S1 Table in S1 File in the SOM. We also pre-registered to test whether 1) social class predicts unethical behavior and 2) whether there is an interaction between the inequality condition and social class in predicting unethical behavior. As these relationships are not the focus of this paper, they are reported in S2 Table in S1 File in the SOM.

### Discussion

Study 1 provided some initial evidence that when people perceive more inequality, they are more likely to expect that they will engage in various unethical behaviors. While the experimental design of the study suggests that this relationship is causal, the nature of the manipulation which asked participants to imagine a hypothetical society, removed the experience from people’s real life. Therefore, in the remaining 3 studies, we addressed this issue by assessing/manipulating perceptions of inequality in people’s state of residence.

## Study 2a

Whereas Study 1 had placed participants in a hypothetical context of either low or high inequality, in Study 2a, we tested whether people’s subjective perceptions of economic inequality in the real world would correlate with their expectations of themselves engaging in unethical behaviors.

### Method

#### Participants

We solicited a convenience sample of Americans on CloudResearch [28]. A sample size of 470 participants allows us to detect a true correlation of .10 with a 95% CI when the corridor of stability is set to a half-width of .10 [34]. We collected data from 581 participants to be above this amount after excluding participants who failed any of the attention checks, and had a final sample of 397 participants (*M* age = 37.50; 52% female; 78% Caucasian, 8% African American, 14% other). A sensitivity power analysis for a two-tailed correlational test with an alpha set to 0.05, 80% power and a sample size of 397 [calculated in G*Power; 29] shows that the minimum effect size for these parameters is *r* = .140.

#### Measures

*Economic inequality*. To assess perceptions of economic inequality, participants completed the Inequality subscale of the 8-item Subjective Inequality Scale on a 7-point Likert scale from “strongly disagree” to “strongly agree” The Inequality subscale measures the extent participants think that economic inequality in their state of residence is high **(***M* = 4.17, *SD* = 1.47; sample item: “Almost all of the money that is earned goes to only a few people”; [35]).

*Unethical behavior*. Participants read the same 10 scenarios from Study 1 (except that they were adapted to apply to society in general rather than in Bimboola) in which they engaged in unethical behavior that they benefitted from (e.g., “You work in a restaurant. It’s against policy to eat food without paying for it. You came straight to your shift from a doctor’s appointment and are therefore hungry. Your supervisor isn’t around, so you make something for yourself and eat it without paying.” Across all 10 scenarios, *M* = 3.60, *SD* = 1.28, α = .85; see SOM for a list of all items).

We also collected variables for a different study that is on an unrelated topic and those are not described here.

### Results

The more participants reported that they perceived economic inequality, the more likely they said they were to engage in unethical behavior, *r*(395) = 0.18, *p* < .001, 95%CI = [0.08, 0.27]. As a robustness check, we reran these analyses with ethnicity, age, and gender as covariates. The results are statistically significant with these covariates and reported in S3 Table in S1 File in the SOM.

### Discussion

These results replicate the finding that perceived inequality is associated with everyday unethical behaviors. Unlike Study 1, this study suggests that this relationship occurs in people’s actual lives. Since we hadn’t pre-registered the study design and hypotheses, we ran a direct pre-registered replication to ensure that the effect is reproducible.

## Study 2b

Study 2b was a direct replication of Study 2a. We pre-registered the hypothesis, measures, exclusion criteria, and analyses on the OSF [https://doi.org/10.17605/OSF.IO/KP4HN].

### Method

#### Participants

We again solicited a convenience sample of Americans on CloudResearch [28]. As pre-registered, based on the lower bound of the 95% CI from Study 3a, we aimed to have a large enough sample to reliably detect a true correlation of .10 at a 95% CI with a corridor of stability of a half-width of .10 [34]; a sample size of at least 470. We collected data from 550 participants to be above this amount after excluding participants for failing one of the two pre-registered attention checks, and had a final sample of 513 participants (*M* age = 38.18; 53% female; 71% Caucasian, 12% Asian, 17% other). A sensitivity power analysis for a two-tailed correlational test with an alpha set to 0.05, 80% power and a sample size of 513 in [calculated in G*Power; 29] shows that the minimum effect size for these parameters is *r* = .123.

### Measures

*Economic inequality*. We used the same measure of subjective inequality as in Study 2a (7-point subjective inequality: *M* = 4.01, *SD* = 1.53, α = .86).

### *Unethical behavior*. We used the same measure of likelihood to engage in unethical behavior as in Study 2a (across 10 scenarios: *M* = 3.45, *SD* = 1.24, α = .85)

Results

First we ran our pre-registered analysis. As hypothesized, the more economic inequality participants reported perceiving, the more likely they said they would engage in various unethical behaviors, *r*(511) = 0.28, *p* < .001, 95%CI = [0.20, 0.36]. As a robustness check, we again reran these analyses with ethnicity, age, and gender as covariates. The results are statistically significant with these covariates and reported in S4 Table in S1 File in the SOM.

### Discussion

These results replicate the results from Study 2a. To summarize, Studies 2a and 2b, provide evidence that the more inequality people perceive, the more likely they are to say they would act unethically. A strength of these two studies is that they suggest that the association between inequality perceptions and self-reported likelihood to act unethically exists in people’s actual lives. However, these two studies were correlational. To further test whether this relationship is causal, we next manipulated people’s perceptions of inequality.

## Study 3

In Study 3, we explored whether informing people (via a video) that the level of inequality in society was high would lead them to say they would behave more unethically in comparison with those who were informed that the level of inequality in society was low. We pre-registered the hypothesis, measures, exclusion criteria, and analyses on the OSF [https://doi.org/10.17605/OSF.IO/7Y4N8].

### Participants

We solicited a convenience sample of Americans on CloudResearch [28]. Given the new manipulation, we estimated an effect size of *d* = .15. A sample of 1100 participants allows us to detect such an effect with 80% power, at an alpha level of .05, with a one-tailed test. To be above this minimum after excluding participants who failed any of the attention checks, we pre-registered to collect data from 1400 participants. We recruited 1437 participants, and after exclusions had a final sample of 1221 participants (*M* age = 36.95; 66% female; 74% Caucasian, 9% African American, 17% other). A sensitivity power analysis for a two-tailed independent samples t-test with an alpha set to 0.05, 80% power and a sample size of 594 in the low, and 627 in the high inequality conditions [calculated in G*Power; 29] shows that the minimum effect size for these parameters is *d* = 0.161.

### Measures

#### Economic inequality

Participants watched a short video that either described that inequality has increased over recent decades or that de facto inequality has dropped because of increases in social spending [24]. To strengthen the effect of the manipulation, after watching the video, participants had to describe in 1–3 sentences how the society they live in is relatively low/high in inequality (low and high inequality conditions, respectively).

#### Unethical behavior

We used the same measure of unethical behavior as in Study 2a (*M* = 3.32, *SD* = 1.26, α = .85).

### Results

As pre-registered, we first tested whether participants in the high inequality condition perceived more inequality in their society than participants in the low inequality condition. We created a mean score of two questions asking to what extent the society participants live in is unequal on a 9-point scale where higher values indicate more inequality. Participants in the high inequality condition (*n* = 627, *M* = 6.25, *SD* = 1.56) perceived significantly more inequality than participants in the low inequality condition (*n* = 594, *M* = 4.87, *SD* = 1.68), *b* = 1.39, *p* < .001, 95%CI = [1.20, 1.57], *d* = 0.85. Next, we tested our main hypothesis. In contrast to our hypothesis, participants in the high inequality condition **(***M* = 3.36, *SD* = 1.28) did not say they would be more likely to act unethically compared with participants in the low inequality condition **(***M* = 3.27, *SD* = 1.24), *b* = 0.09, *p* = .202, 95%CI = [-0.05, 0.23], *d* = 0.07. We also pre-registered to rerun these analyses controlling for different variables and after excluding participants whose description of the video was not in line with the content of the video. The results were still not significant and are reported in S5 Table in S1 File in the SOM. We also pre-registered to test whether 1) social class predicts unethical behavior and 2) whether there is an interaction between the inequality condition and social class in predicting unethical behavior. As these relationships are not the focus of this paper, they are reported in the S6 Table in S1 File in the SOM.

### Discussion

Although the mean difference between the two inequality conditions was in the right direction, we failed to find the hypothesized effect of inequality. This raises the possibility that the effect may not actually exist reliably (and causally) when it is embedded in people’s actual lives. However, it is also more difficult to temporarily shift people’s experience of inequality *and* for that shift to impact people’s self-reported cheating expectations.

Indeed, after the manipulation, we asked participants to describe in 1–3 sentences how the society they live in is either low or high in inequality (depending on the condition they were assigned to). Two coders blind to the hypothesis rated those answers, and as pre-registered we also re-ran the analysis with only people included who gave a response that was sensible (i.e., in line with the condition they were assigned to, as described in the results section, these results are in the SOM). The mean difference became larger but remained non-significant. This suggests that people who didn’t believe the videos weakened the effect and it also suggests that it is possible that some of the people who gave a response in line with the prompt may have followed the instructions but not actually believed the content. While this is a possibility, we have no way of testing it. However, if this is the case, then it would have undermined the manipulation.

## Internal meta-analysis studies 1–3

Since the effects obtained in the 4 studies were not consistently significant, we conducted an internal meta-analysis of them to get a better estimate of the magnitude of this effect. Because We used the estimates for each study that we obtained from the models without covariates as described above and converted them into correlations using the metafor package [36]. A fixed-effect internal meta-analysis confirms that across the 4 studies greater economic inequality is associated with increased expectations for unethical behavior, *r* = .11, *z* = 6.20, *p <* .001, 95%CI = [0.08, 0.15] (S2 Fig).

## General discussion

Economic inequality has been increasing over recent decades, and this is problematic because inequality has been associated with several negative consequences [e.g., 1, 16] including more unethical behaviors [e.g., 4, 6], and the acceptance of unethical behaviors by others [25]. The present research suggests that perceiving high levels of inequality leads people to expect that they will act more unethically.

In Study 1, there was a marginal effect for people who imagined living in a society with high compared to low inequality saying they would be more likely to engage in everyday unethical behavior. In Studies 2a and 2b, people who perceived more inequality, said they would be more likely to engage in everyday unethical behavior. However, in Study 3, participants who saw a video informing them that their society had higher levels of inequality were not significantly more likely to report a willingness to engage in unethical behavior compared with those who were informed that economic inequality was relatively low. An internal meta-analysis across all 4 studies showed a small but significant effect.

### Contributions

The results of this research provide a plausible mechanism for the common finding of past research that higher economic inequality is associated with lower levels of trust [e.g., 37, 38]. As suggested by the present research, people are expecting that they will act more unethically. In turn, this belief may then decrease levels of trust. If people expect that they will act more unethically, societal functioning and cooperation may become undermined [e.g., 25]. Further, this belief may also increase the actual occurrence of unethical behavior [39, 40].

### Limitations and open questions

While we found convergent evidence across different conceptualizations of inequality, the studies also have some limitations. First, we only asked people how likely they would be to engage in unethical behavior, and did not measure actual behavior. Describing various scenarios and asking participants to indicate how likely they would be to act in the ways described, while tapping into people’s expectations of behaving unethically, does not capture their actual behvaior. It is possible that people wrongly assume that they would be more likely to act unethically when inequality is high. Nonetheless, this assumption itself, even if wrong, likely has negative consequences such as undermining trust. Future research would benefit from replicating these results with measures that tap into actual unethical behavior and other potential downstream consequences.

In addition, the scenarios we relied on across all 4 studies were adapted from previous research [25, 33]. While the original scenarios have been validated [33], we have not done so ourselves, and we did not include any checks to ensure that participants understood the content of the scenarios. While we did embed an attention check question within the scenarios that prompted participants to check a particular response, we cannot be certain that all participants understood the scenarios.

Furthermore, Studies 2a and 2b, which yielded the largest effect size, were correlational. While it is possible that, in line with the hypothesis, greater perceptions of inequality caused an increased willingness to engage in unethical behavior, it is also possible that people who are more likely to say they would act unethically perceive society to be more unequal or that some unmeasured alternative variable accounts for the relationship. We proposed that heightened status anxiety and competitiveness may mediate the relationship between perceived inequality and expectations of unethical behavior. Future research could include these mediators to further corroborate the findings of the present research.

When we manipulated perceptions of inequality (Studies 1 and 3), we only included conditions of low and high inequality. While our theorizing hypothesizes that a context of high inequality increases expectations of unethical behavior, it is also possible that a perceived context of low inequality decreases expectations of unethical behavior, or that both of these happen. Future research might benefit by adding a condition that doesn’t provide any information about inequality to compare the high and low inequality contexts against a baseline level of expectations of unethical behavior. In addition, while the manipulation of inequality in Study 3 successfully shifted people’s perceptions of inequality, it did not lead to the hypothesized difference in expected unethical behavior. While this raises the possibility that the relationship might not actually exist in the way hypothesized, it also raises the possibility that the manipulation itself may not be suitable for finding this effect. Specifically, because the video manipulation was embedded in people’s lives and experiences, their beliefs and attitudes about the society they live in may suppress any effects the manipulation itself might have otherwise. Methodologically, this raises the question whether it is useful to manipulate inequality perceptions in the real world, because people already have pre-existing beliefs and attitudes that may be resistant to change. Theoretically, this raises the possibility that part of the reason why objective measures of inequality don’t always yield the same results is because they don’t map perfectly onto people’s perceptions or are suppressed by people’s beliefs about and attitudes towards inequality. For example, research has found that people who believe in social mobility also perceive less inequality and show greater well-being than people who don’t believe in mobility [41–44]. More research is needed to systematically assess to what extent inequality manipulations that shift people’s beliefs about inequality in their society are useful.

Relatedly, in our manipulations of inequality, we didn’t provide any context about how this inequality came to be in the first place. In reality, people likely have assumptions why there is inequality and those assumptions themselves may affect expectations about the likelihood of unethical behavior. For example, if people believe that the society they live in is meritocratic (or came about through fair means), they may be less likely to expect unethical behavior. Future research could investigate how beliefs about the fairness or unfairness of inequality affects expectations of unethical behavior.

We conducted all studies online, and it is possible that the anonymity of an online context makes people more likely to consider engaging in unethical behavior as opposed to an in-person study. Moreover, the studies were conducted exclusively with American samples which limits their generalizability. Specifically, the US has higher levels of inequality than most other industrialized countries, and it is possible that these results wouldn’t replicate in less unequal countries. Furthermore, it is also possible that in less developed countries with higher poverty rates, inequality may be less relevant for unethical behavior. Moreover, it is conceivable that in some cultural contexts, such as in societies with less anonymity, the costs of unethical behavior may outweigh the benefits even at very high levels of inequality. Our measures of unethical behavior focused on financial advantages. It is possible that in societies where status is less dependent on a person’s income, but, for example, on occupational prestige or adherence to religious norms [45], unethical behavior would be more likely to occur in other domains that are more relevant for status.

## Supporting information

S1 Fig Distribution of rewards for the low (left panel) and high (right panel) inequality conditions.
(PNG)

S2 Fig Distribution of rewards for the low (left panel) and high (right panel) inequality conditions. Forest plot showing the correlation between economic inequality and unethical behavior. The size of each square in the forest plot is proportional to the weight of that sample. The estimate for the fixed-effects model is also given. CI = confidence interval.
(PNG)

S1 File (DOCX)
