# Supplementary figures and images for: When are people more open to cheating? Economic inequality makes people expect more everyday unethical behavior
Source: PLoS One. 2024 Feb 21;19(2):e0294124. doi: 10.1371/journal.pone.0294124 (PMC10880980; doi:10.1371/journal.pone.0294124)

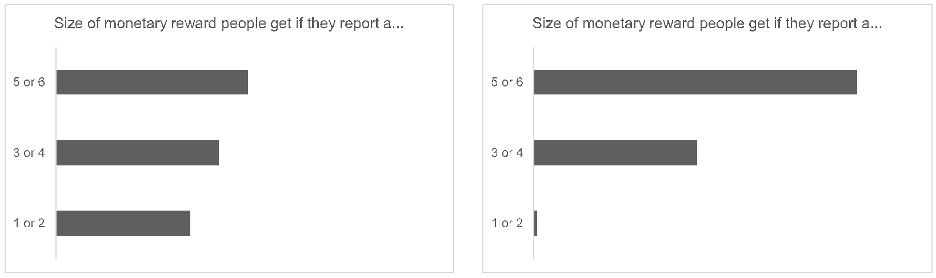

Supplement: S1 Fig — Distribution of rewards for the low (left panel) and high (right panel) inequality conditions. (PNG) [file pone.0294124.s001.png]

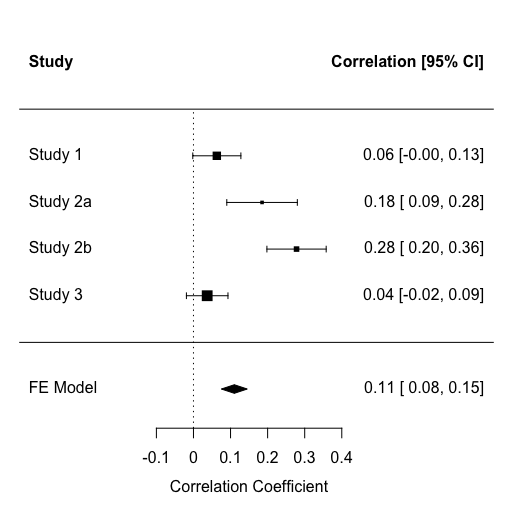

Supplement: S2 Fig — Distribution of rewards for the low (left panel) and high (right panel) inequality conditions. Forest plot showing the correlation between economic inequality and unethical behavior. The size of each square in the forest plot is proportional to the weight of that sample. The estimate for the fixed-effects model is also given. CI = confidence interval. (PNG) [file pone.0294124.s002.png]
