# Supplementary material for: When are people more open to cheating? Economic inequality makes people expect more everyday unethical behavior
Source: PLoS One. 2024 Feb 21;19(2):e0294124. doi: 10.1371/journal.pone.0294124 (PMC10880980; doi:10.1371/journal.pone.0294124)
Supplement: S1 File — (DOCX) [file pone.0294124.s003.docx]

**Study 1**

**Unethical Behavior Vignettes**

1. “You work in a fast-food restaurant in downtown Bimboola. It's against policy to eat food without paying for it. You came straight from a doctor's appointment and are therefore hungry. Your supervisor isn't around, so you make something for yourself and eat it without paying.”

2. “You work as an office assistant for a large Bimboolean corporation. You're alone in the office making copies and realize you're out of copy paper at home. You therefore slip a ream of paper into your bag.”

3. “You are a student at the University of Bimboola. You are preparing for the final examination of a 'Creative Writing' course. Some of your friends have somehow obtained the writing prompt that will be part of the final exam. You ask them for the prompt and plan your essay out accordingly.”

4. “You've waited in line for 10 minutes to buy a coffee and muffin at a coffee shop in downtown Bimboola. When you're a couple of blocks away, you realize that the clerk gave you change for 20 Bimboolean Dollars rather than for the 10 Bimboolean Dollars you gave him. You savor your coffee, muffin and free 10 Bimboolean Dollars.”

5. “You are a student at the University of Bimboola. You get the final examination back from your professor and notice that he's marked correct three answers that you got wrong. Revealing these errors would mean the difference between an A and a B grade. You say nothing.”

6. “Your accounting course requires you to purchase a software package that sells for 50 Bimboolean Dollars. Your friend, who is also in the course, has already bought the software and offers to lend it to you. Even though this is not allowed, you take it and load it onto your computer.”

7. “Your boss asks you to get confidential information about a competitor's product. You therefore pose as a university student doing a research project on the competitor's company and ask for the information.”

8. “You are a student at the University of Bimboola. You are assigned a team project in one of your courses. Your team waits until the last minute to begin working. Several team members suggest using an old project out of their fraternity/sorority files. You go along with this plan.”

9. “You are checking your bank account balance online and notice that a transfer of 50 Bimboolean Dollars to your account had been made a week ago. You do not recall anyone owing you that sum of money but you say nothing to the bank about it.”

10. “You receive a package at your doorstep from a trendy fashion store one day. You are not expecting any gifts or online orders from this store. You realize that the package was addressed to the previous tenant. Inside the package is a pair of expensive looking pants that fit you perfectly. You keep them for yourself.”

**S1 Table.** Relationship between economic inequality and unethical behavior with age, gender, and ethnicity as covariates

|  | **Everyday unethical behavior** | | |
| --- | --- | --- | --- |
| *Predictors* | *Estimates* | *CI* | *p* |
| (Intercept) | 4.337 | 4.066 – 4.608 | **<0.001** |
| Condition  (0=low inequality,  1=high inequality) | 0.179 | 0.025 – 0.334 | **0.023** |
| Age | -0.026 | -0.032 – -0.020 | **<0.001** |
| Gender  (0=female, 1=male) | 0.114 | -0.046 – 0.274 | 0.163 |
| Black/African American | -0.311 | -0.587 – -0.036 | **0.027** |
| Asian | -0.145 | -0.450 – 0.160 | 0.350 |
| Hispanic | 0.276 | -0.057 – 0.610 | 0.104 |
| Other Ethnicity | -0.077 | -0.539 – 0.385 | 0.743 |
| Observations | 901 | | |
| R^2^ / R^2^ adjusted | 0.099 / 0.092 | | |

*Note.* Ethnicities are dummy coded; White/Caucasian is the reference group.

|  |  |  |  |
| --- | --- | --- | --- |

**S2 Table.** Additional pre-registered analyses predicting unethical behavior from social class and an interaction between social class and inequality condition

|  | **Everyday unethical behavior** | | | **Everyday unethical behavior** | | | **Everyday unethical behavior** | | | **Everyday unethical behavior** | | | **Everyday unethical behavior** | | | **Everyday unethical behavior** | | |
| --- | --- | --- | --- | --- | --- | --- | --- | --- | --- | --- | --- | --- | --- | --- | --- | --- | --- | --- |
| *Predictors* | *Estimates* | *CI* | *p* | *Estimates* | *CI* | *p* | *Estimates* | *CI* | *p* | *Estimates* | *CI* | *p* | *Estimates* | *CI* | *p* | *Estimates* | *CI* | *p* |
| (Intercept) | 3.64 | 3.37 – 3.91 | **<0.001** | 4.61 | 4.25 – 4.97 | **<0.001** | 3.72 | 3.34 – 4.11 | **<0.001** | 3.65 | 3.36 – 3.94 | **<0.001** | 4.61 | 4.24 – 4.98 | **<0.001** | 3.62 | 3.22 – 4.03 | **<0.001** |
| Subjective socioeconomic status (SES) | -0.04 | -0.09 – 0.01 | 0.102 | -0.04 | -0.08 – 0.01 | 0.142 | -0.07 | -0.14 – -0.00 | **0.044** |  |  |  |  |  |  |  |  |  |
| Social Class |  |  |  | -0.03 | -0.03 – -0.02 | **<0.001** |  |  |  |  |  |  | -0.03 | -0.03 – -0.02 | **<0.001** |  |  |  |
| Age |  |  |  | 0.10 | -0.06 – 0.26 | 0.235 |  |  |  |  |  |  | 0.10 | -0.06 – 0.26 | 0.227 |  |  |  |
| Gender  (0=female, 1=male) |  |  |  | -0.32 | -0.59 – -0.04 | **0.024** |  |  |  |  |  |  | -0.33 | -0.61 – -0.05 | **0.020** |  |  |  |
| Black/African American |  |  |  | -0.15 | -0.45 – 0.16 | 0.345 |  |  |  |  |  |  | -0.14 | -0.45 – 0.16 | 0.357 |  |  |  |
| Asian |  |  |  | 0.28 | -0.06 – 0.61 | 0.106 |  |  |  |  |  |  | 0.27 | -0.06 – 0.61 | 0.111 |  |  |  |
| Hispanic |  |  |  | -0.11 | -0.57 – 0.35 | 0.643 |  |  |  |  |  |  | -0.12 | -0.59 – 0.34 | 0.608 |  |  |  |
| Other Ethnicity |  |  |  |  |  |  | -0.17 | -0.71 – 0.36 | 0.526 |  |  |  |  |  |  | 0.00 | -0.57 – 0.58 | 0.987 |
| Condition  (0=low inequality,  1=high inequality) |  |  |  |  |  |  | 0.06 | -0.03 – 0.16 | 0.203 |  |  |  |  |  |  |  |  |  |
| SES * Condition |  |  |  |  |  |  |  |  |  | -0.08 | -0.19 – 0.02 | 0.117 | -0.07 | -0.17 – 0.03 | 0.173 | -0.10 | -0.25 – 0.04 | 0.166 |
| Social Class * Condition |  |  |  |  |  |  |  |  |  |  |  |  |  |  |  | 0.06 | -0.15 – 0.27 | 0.601 |
| Observations | 901 | | | 901 | | | 901 | | | 901 | | | 901 | | | 901 | | |
| R^2^ / R^2^ adjusted | 0.003 / 0.002 | | | 0.096 / 0.089 | | | 0.009 / 0.006 | | | 0.003 / 0.002 | | | 0.095 / 0.088 | | | 0.007 / 0.004 | | |

*Note.* Ethnicities are dummy coded; White/Caucasian is the reference group. Subjective socioeconomic status was measured on a ladder with 10 rungs that indicated one’s relative standing in society (*M*=5.19, *SD*=1.64; 1); for social class participants indicated which of five social classes (i.e., poor, working class, middle class, upper-middle class, and upper class) they thought they belonged to (*M*=2.63, *SD*=0.77; 2).

**Study 2a**

**Unethical Behavior Vignettes**

1. “You work in a restaurant. It’s against policy to eat food without paying for it. You came straight to your shift from a doctor’s appointment and are therefore hungry. Your supervisor isn’t around, so you make something for yourself and eat it without paying.”

2. “You work as an office assistant for a large corporation. You’re alone in the office making copies and realize you’re out of copy paper at home. You therefore slip a ream of paper into your bag.”

3. “You are a university student preparing for the final examination of a 'Creative Writing' course. Some of your friends have somehow obtained the writing prompt that will be part of the final exam. You ask them for the prompt and plan your essay out accordingly.”

4. “You’ve waited in line for 10 minutes to buy a coffee and muffin at a coffee shop. When you’re a couple of blocks away, you realize that the clerk gave you change for $20 rather than for the $10 you gave him. You savor your coffee, muffin and free $10.”

5. “You have completed a test for a routine safety course at work. When you receive your test results back, you notice that your supervisor has marked correct three answers that you got wrong. Revealing these errors would mean the difference between you passing and having to study for the test all over again to retake it next month. You say nothing.”

6. “Your accounting course requires you to purchase a software package that sells for $50. Your friend, who is also in the course, has already bought the software and offers to lend it to you. Even though this is not allowed, you take it and load it onto your computer.”

7. “Your boss asks you to get confidential information about a competitor’s product. You therefore pose as a researcher doing a study on the competitor’s company and ask for the information.”

8. “You own a high-quality counterfeit designer wallet that you are trying to sell online. Someone mistakenly thinks that the wallet is actual brand-name and offers you a lot of money for it. After realizing the misunderstanding, you say nothing and allow them to overpay for the wallet.”

9. “You are checking your bank account balance online and notice that a transfer of $50 to your account had been made a week ago. You do not recall anyone owing you that sum of money but you say nothing to the bank about it.”

10. “You receive a package at your doorstep from a trendy fashion store one day. You are not expecting any gifts or online orders from this store. You realize that the package was addressed to the previous tenant. Inside the package is a pair of expensive looking pants that fit you perfectly. You keep them for yourself.”

**S3 Table.** Association between subjective inequality and unethical behavior controlling for age, gender, and ethnicity

|  | **Everyday unethical behavior** | | |
| --- | --- | --- | --- |
| *Predictors* | *Estimates* | *CI* | *p* |
| (Intercept) | 4.55 | 3.91 – 5.19 | **<0.001** |
| Subjective Inequality | 0.10 | 0.02 – 0.18 | **0.016** |
| Age | -0.04 | -0.05 – -0.03 | **<0.001** |
| Gender  (0=female, 1=male) | 0.04 | -0.20 – 0.28 | 0.743 |
| Black/African American | 0.07 | -0.36 – 0.50 | 0.744 |
| Asian | 0.63 | 0.09 – 1.16 | **0.023** |
| Hispanic | 0.02 | -0.46 – 0.50 | 0.940 |
| Other Ethnicity | -1.38 | -2.27 – -0.49 | **0.003** |
| Observations | 395 | | |
| R^2^ / R^2^ adjusted | 0.166 / 0.151 | | |

*Note.* Ethnicities are dummy coded; White/Caucasian is the reference group.

**Study 2b**

**S4 Table.** Association between subjective inequality and unethical behavior controlling for age, gender, and ethnicity

|  | **Everyday unethical behavior** | | |
| --- | --- | --- | --- |
| *Predictors* | *Estimates* | *CI* | *p* |
| (Intercept) | 3.49 | 3.02 – 3.96 | **<0.001** |
| Subjective Inequality | 0.18 | 0.11 – 0.25 | **<0.001** |
| Age | -0.02 | -0.03 – -0.02 | **<0.001** |
| Gender  (0=female, 1=male) | 0.24 | 0.03 – 0.44 | **0.022** |
| Black/African American | -0.21 | -0.61 – 0.18 | 0.292 |
| Asian | -0.01 | -0.33 – 0.30 | 0.930 |
| Hispanic | 0.43 | 0.04 – 0.82 | **0.030** |
| Other Ethnicity | -0.04 | -0.76 – 0.69 | 0.918 |
| Observations | 507 | | |
| R^2^ / R^2^ adjusted | 0.162 / 0.150 | | |

*Note.* Ethnicities are dummy coded; White/Caucasian is the reference group.

**Study 3**

**S5 Table.** Pre-registered analyses with additional pre-registered covariates and exclusion criteria

|  | **Everyday unethical behavior**  **Model 1** | | | **Everyday unethical behavior**  **Model 2** | | | **Everyday unethical behavior**  **Model 3** | | | **Everyday unethical behavior**  **Model 4** | | | **Everyday unethical behavior**  **Model 5** | | | **Everyday unethical behavior**  **Model 6** | | |
| --- | --- | --- | --- | --- | --- | --- | --- | --- | --- | --- | --- | --- | --- | --- | --- | --- | --- | --- |
| *Predictors* | *Estimates* | *CI* | *p* | *Estimates* | *CI* | *p* | *Estimates* | *CI* | *p* | *Estimates* | *CI* | *p* | *Estimates* | *CI* | *p* | *Estimates* | *CI* | *p* |
| (Intercept) | 4.17 | 3.93 – 4.42 | **<0.001** | 3.80 | 3.48 – 4.11 | **<0.001** | 4.61 | 4.34 – 4.88 | **<0.001** | 4.25 | 3.98 – 4.52 | **<0.001** | 3.89 | 3.54 – 4.23 | **<0.001** | 4.75 | 4.44 – 5.05 | **<0.001** |
| Condition  (0=low inequality,  1=high inequality) | 0.08 | -0.06 – 0.22 | 0.259 | 0.03 | -0.11 – 0.17 | 0.701 | 0.07 | -0.06 – 0.21 | 0.306 | 0.11 | -0.05 – 0.26 | 0.168 | 0.03 | -0.13 – 0.19 | 0.734 | 0.06 | -0.09 – 0.21 | 0.428 |
| Age | -0.03 | -0.03 – -0.02 | **<0.001** | -0.03 | -0.03 – -0.02 | **<0.001** | -0.02 | -0.03 – -0.02 | **<0.001** | -0.03 | -0.03 – -0.02 | **<0.001** | -0.03 | -0.03 – -0.02 | **<0.001** | -0.02 | -0.03 – -0.02 | **<0.001** |
| Gender  (0=female, 1=male) | 0.10 | -0.04 – 0.25 | 0.169 | 0.11 | -0.04 – 0.25 | 0.153 | 0.16 | 0.01 – 0.30 | **0.032** | 0.07 | -0.10 – 0.23 | 0.432 | 0.06 | -0.10 – 0.23 | 0.437 | 0.13 | -0.03 – 0.29 | 0.103 |
| Black/African American | 0.10 | -0.15 – 0.34 | 0.444 | 0.03 | -0.21 – 0.28 | 0.790 | 0.04 | -0.21 – 0.28 | 0.761 | 0.01 | -0.28 – 0.29 | 0.969 | -0.05 | -0.34 – 0.23 | 0.725 | -0.07 | -0.35 – 0.21 | 0.631 |
| Asian | -0.10 | -0.38 – 0.19 | 0.505 | -0.11 | -0.39 – 0.17 | 0.455 | -0.10 | -0.38 – 0.18 | 0.474 | 0.06 | -0.26 – 0.37 | 0.723 | 0.06 | -0.25 – 0.37 | 0.695 | 0.03 | -0.28 – 0.34 | 0.842 |
| Hispanic | 0.15 | -0.14 – 0.43 | 0.318 | 0.13 | -0.16 – 0.42 | 0.372 | 0.01 | -0.27 – 0.30 | 0.920 | 0.04 | -0.29 – 0.37 | 0.811 | 0.05 | -0.28 – 0.37 | 0.776 | -0.10 | -0.42 – 0.22 | 0.543 |
| Other Ethnicity | -0.03 | -0.38 – 0.32 | 0.870 | -0.07 | -0.42 – 0.27 | 0.677 | -0.06 | -0.40 – 0.28 | 0.745 | -0.06 | -0.43 – 0.31 | 0.753 | -0.12 | -0.49 – 0.26 | 0.543 | -0.10 | -0.47 – 0.26 | 0.575 |
| Unfairness of Inequality |  |  |  | 0.08 | 0.03 – 0.12 | **<0.001** |  |  |  |  |  |  | 0.08 | 0.03 – 0.12 | **0.001** |  |  |  |
| Conservatism |  |  |  |  |  |  | -0.15 | -0.19 – -0.10 | **<0.001** |  |  |  |  |  |  | -0.16 | -0.21 – -0.12 | **<0.001** |
| Observations | 1209 | | | 1209 | | | 1209 | | | 976 | | | 976 | | | 976 | | |
| R^2^ / R^2^ adjusted | 0.074 / 0.069 | | | 0.084 / 0.078 | | | 0.107 / 0.101 | | | 0.084 / 0.078 | | | 0.095 / 0.087 | | | 0.124 / 0.117 | | |

*Note.* Ethnicities are dummy coded; White/Caucasian is the reference group. Unfairness of Inequality: higher score = inequality is perceived as more unfair. Models 1-3: Controlling for different covariates; Models 4-6: Controlling for different covariates after excluding participants who failed to provide a sensible response to a prompt asking them to describe how the society they live in is low (for the low inequality condition) or high (for the high inequality condition) in inequality.

**S6 Table.** Additional pre-registered analyses predicting unethical behavior from social class and an interaction between social class and inequality condition

|  | **Everyday unethical behavior**  **Model 1** | | | **Everyday unethical behavior**  **Model 2** | | | **Everyday unethical behavior**  **Model 3** | | | **Everyday unethical behavior**  **Model 4** | | | **Everyday unethical behavior**  **Model 5** | | | **Everyday unethical behavior**  **Model 6** | | | **Everyday unethical behavior**  **Model 7** | | | **Everyday unethical behavior**  **Model 8** | | |
| --- | --- | --- | --- | --- | --- | --- | --- | --- | --- | --- | --- | --- | --- | --- | --- | --- | --- | --- | --- | --- | --- | --- | --- | --- |
| *Predictors* | *Estimates* | *CI* | *p* | *Estimates* | *CI* | *p* | *Estimates* | *CI* | *p* | *Estimates* | *CI* | *p* | *Estimates* | *CI* | *p* | *Estimates* | *CI* | *p* | *Estimates* | *CI* | *p* | *Estimates* | *CI* | *p* |
| (Intercept) | 3.44 | 3.22 – 3.66 | **<0.001** | 4.48 | 4.16 – 4.81 | **<0.001** | 3.24 | 3.00 – 3.47 | **<0.001** | 4.30 | 3.95 – 4.65 | **<0.001** | 3.24 | 2.89 – 3.58 | **<0.001** | 3.07 | 2.71 – 3.43 | **<0.001** | 3.23 | 2.82 – 3.65 | **<0.001** | 3.05 | 2.60 – 3.50 | **<0.001** |
| Subjective  socioeconomic  status (SES) | -0.02 | -0.07 – 0.02 | 0.255 | -0.03 | -0.08 – 0.01 | 0.127 |  |  |  |  |  |  | 0.01 | -0.06 – 0.07 | 0.835 |  |  |  | 0.00 | -0.07 – 0.08 | 0.922 |  |  |  |
| Social Class |  |  |  |  |  |  | 0.03 | -0.06 – 0.12 | 0.487 | 0.01 | -0.09 – 0.10 | 0.861 |  |  |  | 0.07 | -0.05 – 0.20 | 0.254 |  |  |  | 0.08 | -0.08 – 0.23 |  |
| Age |  |  |  | -0.03 | -0.03 – -0.02 | **<0.001** |  |  |  | -0.03 | -0.03 – -0.02 | **<0.001** |  |  |  |  |  |  |  |  |  |  |  |  |
| Gender  (0=female,  1=male) |  |  |  | 0.07 | -0.10 – 0.23 | 0.425 |  |  |  | 0.06 | -0.10 – 0.22 | 0.484 |  |  |  |  |  |  |  |  |  |  |  |  |
| Black/  African American |  |  |  | 0.00 | -0.28 – 0.29 | 0.981 |  |  |  | 0.01 | -0.28 – 0.29 | 0.965 |  |  |  |  |  |  |  |  |  |  |  |  |
| Asian |  |  |  | 0.07 | -0.24 – 0.38 | 0.667 |  |  |  | 0.05 | -0.26 – 0.37 | 0.735 |  |  |  |  |  |  |  |  |  |  |  |  |
| Hispanic |  |  |  | 0.05 | -0.28 – 0.37 | 0.779 |  |  |  | 0.05 | -0.28 – 0.38 | 0.771 |  |  |  |  |  |  |  |  |  |  |  |  |
| Other Ethnicity |  |  |  | -0.07 | -0.44 – 0.30 | 0.717 |  |  |  | -0.06 | -0.44 – 0.31 | 0.739 |  |  |  |  |  |  |  |  |  |  |  |  |
| Condition  (0=low inequality,  1=high inequality) |  |  |  |  |  |  |  |  |  |  |  |  | 0.35 | -0.10 – 0.80 | 0.128 | 0.29 | -0.19 – 0.77 | 0.243 | 0.49 | -0.03 – 1.01 | 0.067 | 0.40 | -0.15 – 0.96 | 0.156 |
| SES * Condition |  |  |  |  |  |  |  |  |  |  |  |  | -0.05 | -0.14 – 0.03 | 0.226 |  |  |  | -0.07 | -0.17 – 0.02 | 0.145 |  |  |  |
| Social Class * Condition |  |  |  |  |  |  |  |  |  |  |  |  |  |  |  | -0.07 | -0.25 – 0.10 | 0.419 |  |  |  | -0.10 | -0.30 – 0.10 | 0.328 |
| Observations | 1221 | | | 976 | | | 1221 | | | 976 | | | 1221 | | | 1221 | | | 976 | | | 976 | | |
| R^2^ / R^2^ adjusted | 0.001 / 0.000 | | | 0.085 / 0.078 | | | 0.000 / -0.000 | | | 0.083 / 0.076 | | | 0.003 / 0.001 | | | 0.002 / -0.000 | | | 0.008 / 0.005 | | | 0.004 / 0.001 | | |

*Note.* Ethnicities are dummy coded; White/Caucasian is the reference group. Subjective socioeconomic status was measured on a ladder with 10 rungs that indicated one’s relative standing in society (*M*=5.09, *SD*=1.70; 1); for social class participants indicated which of five social classes (i.e., poor, working class, middle class, upper-middle class, and upper class) they thought they belonged to (*M*=2.59, *SD*=0.81; 2). Models 7-8: Excluding participants who failed to provide a sensible response to a prompt asking them to describe how the society they live in is low (for the low inequality condition) or high (for the high inequality condition) in inequality.

References

1. Adler NE, Epel ES, Castellazzo G, Ickovics JR. Relationship of subjective and objective social status with psychological and physiological functioning: Preliminary data in healthy, White women. Health Psychol [Internet]. 2000;19(6):586–92. Available from: <http://dx.doi.org/10.1037/0278-6133.19.6.586>
2. Jackman MR, Jackman RW. Class awareness in the United States. Berkeley: University of California Press; 1983.
